# Clinical and biochemical parameters associated with substance-induced psychotic disorder: which differences between alcohol, cannabis and psychostimulants

**DOI:** 10.1192/j.eurpsy.2023.1092

**Published:** 2023-07-19

**Authors:** G. Nosari, A. Ceresa, M. Di Paolo, C. M. Esposito, V. Ciappolino, A. Calabrese, F. Legnani, A. M. Auxilia, M. Capellazzi, I. Tagliabue, L. Cirella, E. Capuzzi, A. Caldiroli, A. Dakanalis, M. Clerici, M. Buoli

**Affiliations:** 1 Department of Pathophysiology and Transplantation; 2 Department of Neurosciences and Mental Health, Fondazione IRCCS Ca’ Granda Ospedale Maggiore Policlinico, University of Milan, Milan; 3 Department of Medicine and Surgery, University of Milano Bicocca, University of Milan Bicocca, Monza; 4 Healthcare Professionals Department Fondazione IRCCS Ca’ Granda Ospedale Maggiore Policlinico, University of Milan, Milan; 5 Psychiatric Department, Azienda Socio-Sanitaria Territoriale Monza, Monza, Italy

## Abstract

**Introduction:**

According to DSM V, substance-induced psychotic disorder is a mental health condition in which the onset of psychotic symptoms can be traced to the use of a psychotropic substance. The pathogenesis of this disease is still poorly understood; current literature traces its causes back to genetic predisposition and early traumatic events (i.e. child abuse).

**Objectives:**

The present study aims to identify specific clinical features and biochemical markers which could be addressed as predictors for the long-term prognosis of this disease. Moreover, we aim to identify specific correlations between the clinical phenotype and the underlying substance abuse, in order to allow the early start of a tailored treatment.

**Methods:**

Between 2020 and 2022 we recruited 218 patients referring to the Policlinico Hospital in Milan and the San Gerardo Hospital in Monza, Italy. All the patients were diagnosed with substance induced psychotic disorder: 31 reported alcohol abuse (14,2%), 71 psichostimulants (32,6%), 116 cannabis, (53,2%). For each patients, we collected demographic data, medical records and a comprehensive psychometric assessment (GAF, PANSS, BPRS, Modified Sad Person Scale-MOAS). Furthermore, we collected a blood sample for dosing Na+, K+, Na+/K+, hemogram with formula and platelets, glucose , urea, creatinine, uric acid, transaminases, γGT, bilirubin, plasma proteins, albumin, LDH, CPK, PCHE, cholesterol, HDL, LDL, Tg, TSH.

**Results:**

Chi squared test (χ²) has been used to compare qualitative variables between the 3 subgrous (alcohol-, psychostimulants- and cannabis-induced psychotic syndromes) (fig.1). One way ANOVA test has been used to compare quantitative variables between the same 3 subgroups (fig.2). After removing one of the subgropus (alcohol-induced psychotic symptoms), the same analysis have been repeated. Significant variables have been included in a binary logistic regression model in order to confirm their validity as predictors for cannabis- and psychostimulants-induced psychotic disorders (fig 3). Finally we performed Omnibus test and Hosmer-Lemeshow test in order to verify the validity of these regression models.

**Image:**

**Figure:**
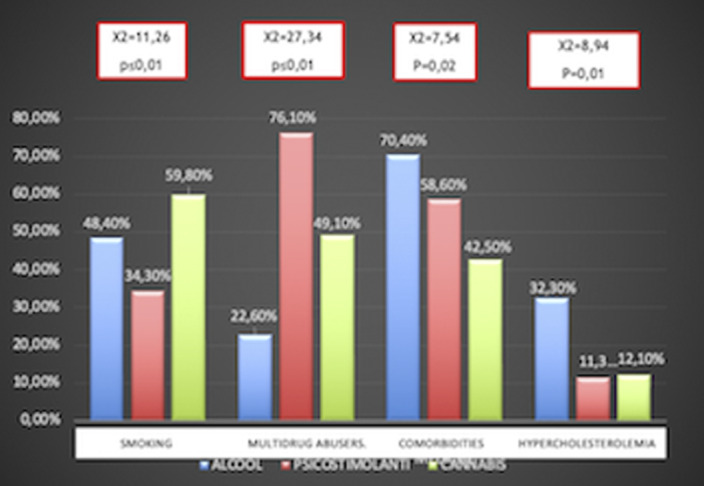

**Image 2:**

**Figure:**
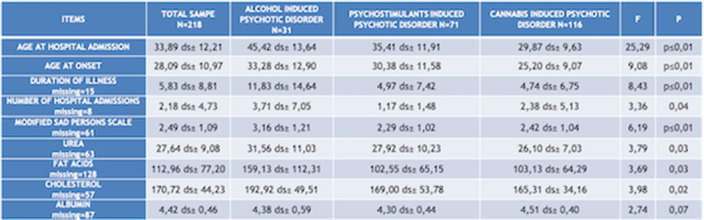

**Image 3:**

**Figure:**
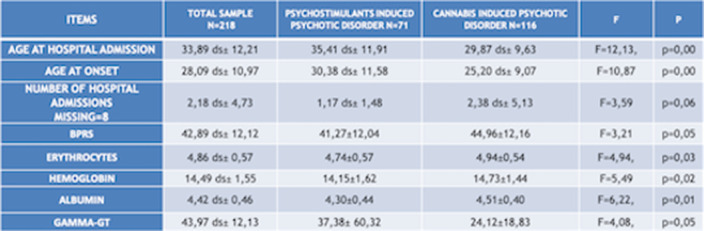

**Conclusions:**

For each considered subgroups, we indentified the following features. Alcohol induced psychotic syndrome: higher age of onset and age of hospital admission, higher cholesterol and hurea levels, , high comorbidity with medical conditions anxiety/depression, low social functioning, higher suicidal risk;, higher hospitalization rate. Cannabis induced psychotic syndrome: higher hemoglobin and albumin levels, more severe psychiatric symtoms (BPRS), higher smoking rates. Psychostimulants induced psychotic syndrome: higher multi-drug abuse risk. We could assume that according to this consideration the treatment protocols for each of these subgroups should be tailored according to their specific features.

**Disclosure of Interest:**

None Declared

